# The Histone Deacetylase Inhibitor BML-210 Influences Gene and Protein Expression in Human Promyelocytic Leukemia NB4 Cells via Epigenetic Reprogramming

**DOI:** 10.3390/ijms160818252

**Published:** 2015-06-06

**Authors:** Veronika Borutinskaitė, Rūta Navakauskienė

**Affiliations:** Department of Molecular Cell Biology, Institute of Biochemistry, Vilnius University, Mokslininkų 12, Vilnius LT 08662, Lithuania; E-Mail: veronika.borutinskaite@bchi.vu.lt

**Keywords:** leukemia, HDAC, BML-210, ATRA, granulocytic differentiation

## Abstract

Today, cancer is understood as an epigenetic as well as genetic disease. The main epigenetic hallmarks of the cancer cell are DNA methylation and histone modifications. Proteins such as histone deacetylases (HDACs) that cause modifications of histones and other proteins can be targets for novel anticancer agents. Recently, interest in compounds that can inhibit HDACs increased, and now there are many HDACs inhibitors (HDACIs) available with different chemical structures, biological and biochemical properties; hopefully some of them will succeed, probably in combination with other agents, in cancer therapies. In our study we focused on the novel HDACI–BML-210. We found that BML-210 (*N*-phenyl-*N*ʹ-(2-Aminophenyl)hexamethylenediamide) inhibits the growth of NB4 cells in dose- and time-dependent manner. In this study we also examined how expression and activity of HDACs are affected after leukemia cell treatment with BML-210. Using a mass spectrometry method we identified proteins that changed expression after treatment with BML-210. We prepared RT-PCR analysis of these genes and the results correlated with proteomic data. Based on these and other findings from our group, we suggest that HDACIs, like BML-210, can be promising anticancer agents in promyelocytic leukemia treatment.

## 1. Introduction

Leukemia is the manifestation of the inappropriate expansion of hematopoietic progenitor cells, often due to a block of cell maturation at early stages in the lineages that give rise to the various cell types that constitute normal blood [1]. White blood cells come from two cell lines: myeloid and lymphoid. Acute myeloid leukemia (AML) is a malignant tumor of hematopoietic precursor cells of myeloid lineage, with an annual incident of 1/10,000 and a frequency that increases with age [1]. Acute promyelocytic leukemia (APL) is a type of AML and accounts for approximately 10% of cases of AML [2]. The main genetic feature (90% APL cases) of this disease is chromosomal translocation t(15;17) (q22;q12) that generates the PML-RARα (promyelocytic leukemia-retinoic acid receptor α) fusion protein comprising parts of the PML and retinoic acid receptor α (*RARα*) genes [1,3]. Normally RARα regulates gene expression through a co-repressor complex containing histone deacetylase (HDAC) activities that induces chromatin condensation and transcriptional repression [4]. When mutated PML-RARα protein forms, it does not respond to its natural ligand all-trans retinoic acid (ATRA) allowing uncontrolled cells proliferation at the promyelocyte stage [5]. However, pharmacological doses (10^−6^ M) of ATRA can induce differentiation of AML cells to metamyelocytes and polymorphonuclear leukocytes [6]. And now such concentrations of ATRA are used to treat patients in so-called differentiation therapy. This therapy is the way to treat cancer using drugs that participate in cell signaling and epigenetic processes and can stop uncontrolled cells proliferation and induce differentiation [7]. To treat APL together with ATRA HDACs inhibitors (HDACI) are used. HDACs are one of the most important proteins, together with histone acetyltransferases (HAT), participating in chromatin remodeling. It is know that the aberrant transcription of genes that control the cell cycle, differentiation and apoptosis is due to altered expression or mutations of genes that encode HAT and HDAC, or their binding and recruiting partners. These changes are important in cancer formation and progression [8]. Histone deacetylase inhibitors are a class of targeted anticancer agents, which are potent inducers of growth arrest, differentiation and/or apoptotic cell death of transformed cells *in vitro* and *in vivo* [9]. HDACI derive from both natural sources (e.g., sodium butyrate, Trichostatin A) and from synthetic routes [10].

In our research we used HDAC inhibitor BML-210 which belongs to synthetic benzamides. In our previous studies we have showed that BML-210 inhibits the growth of NB4, HL-60, THP-1 and K562 cell lines and promotes apoptosis in a dose- and time-dependent manner and also alone induces HL-60 and K562 cell differentiation [11].

In this study we investigated how BML-210 treatment affects growth, viability and apoptosis of promyelocytic leukemia cells (NB4) and how expression and activity of HDACs are influenced by HDAC inhibitor BML-210. We found that BML-210 inhibits the growth of NB4 cell lines and promotes apoptosis in a dose- and time-dependent manner. This correlated with cell cycle arrest at the G0/G1 stage. BML-210 inhibited HDACs activity as well as the expression of HDAC1 in NB4 cells. Using a mass spectrometry method we identified proteins that changed expression after treatment with BML-210. We prepared RT-PCR analysis of these genes and the results correlated with proteomic data. We showed that after BML-210 treatment, endoplasmin, calreticulin, 14-3-3 protein eta, and proliferating cell nuclear antigen were down-regulated, while a few proteins were up-regulated: chloride intracellular channel protein 1, lactoylglutathione lyase, *etc*. All these proteins are important in cancer cell proliferation and apoptosis.

Based on our findings, we suggest that HDACIs, like BML-210, can be promising anticancer agents in promyelocytic leukemia treatment.

## 2. Results

### 2.1. Influence of BML-210 on NB4 Cell Growth, Viability, Cell Cycle Progression and Apoptosis

**Figure 1:**
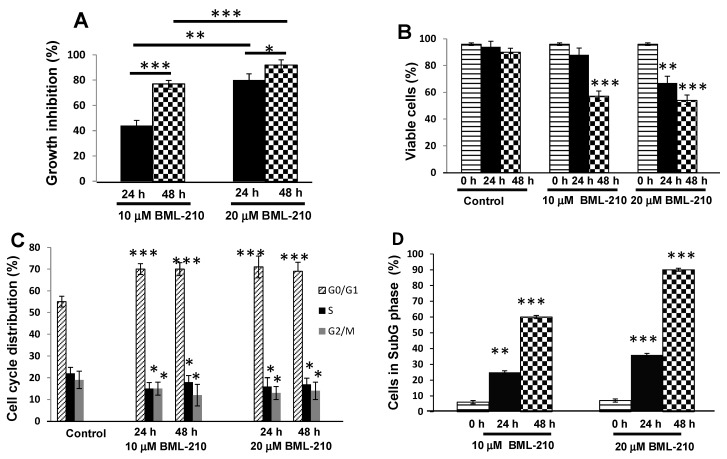

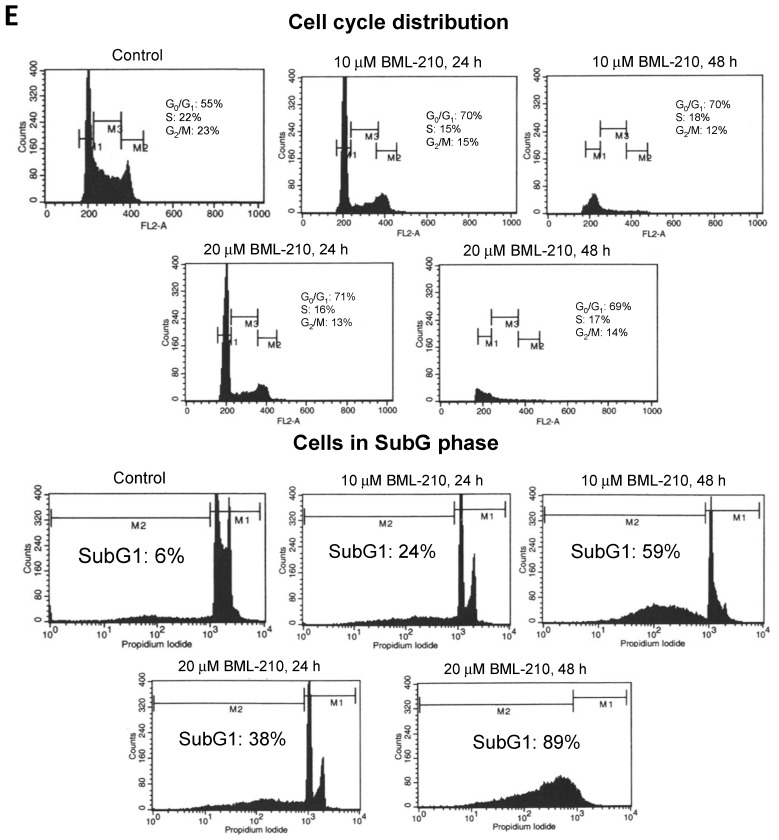
Effects of BML-210 on NB4 cell growth inhibition, survival and cell cycle distribution. Cells were treated with BML-210 at the indicated concentrations for three days. (**A**) The percentage of cell growth inhibition; (**B**) Viable cell number was determined daily by counting in a hemocytometer after staining with 0.2% trypan blue; (**C**) Flow cytometric analysis of cell cycle distribution between G0/G1, S and G2/M phases (%) after treatments. Representative FACS-generated histograms of a hypodiploid peak (subG1) in control cells and after treatment with BML-210 for 24 and 48 h (presented in (**E**)); (**D**) Apoptosis was determined as a hypodiploid peak (subG1) from flow cytometric analysis of PI stained cells. Apoptosis is presented as a percentage of the total events collected. Representative FACS-generated histograms of a hypodiploid peak (subG1) in control cells and after treatment with BML-210 for 24, 48 h (presented in (**E**)). Results are mean ± SEM (*n* = 3). * *p* < 0.05, ** *p* < 0.001 and *** *p* < 0.0001.

NB4 cells were incubated with 10 and 20 μM BML-210 and analyzed for growth inhibition and cell cycle distribution by flow cytometry. BML-210 inhibited cell proliferation and growth inhibition of NB4 cells in a dose- and time-dependent manner (Figure 1A,B). We determined that 10 μM BML-210 inhibited growth of NB4 cells up to 44% and 77% at day one and two, respectively (Figure 1A). The 10 μM BML-210 treatment also caused anti-proliferative effect: the viable cell number in culture was 88% (after 24 h) and 57% (after 48 h) (Figure 1B). An even stronger effect was observed after treatment with 20 μM BML-210: 67% of viable cells were detected at 24 h point of treatment and growth inhibition up to 90% at 48 h point (Figure 1A,B).

Cell cycle analysis revealed that BML-210 caused a decrease in the proportion of NB4 cells in the S phase and an increase in the G0/G1 phase (Figure 1C). Ten μM BML-210 alone caused an increase in the G0/G1 phase up to 70% at 24 and 48 h (Figure 1C). The higher dose of BML-210 (20 μM) showed similar effects on cell cycle progression and caused an increase in the G0/G1 phase up to 71% at 24 h and 69% at 48 h (Figure 1C).

BML-210 at a concentration up to 20 μM caused cytotoxic effects on NB4 cells in a dose- and time-dependent manner, as shown in Figure 1D. BML-210 at a dose of 10 μM induced apoptotic cell death (to 60%) as was determined by flow cytometry after cell staining with PI on day two (Figure 1D) and 20 μM concentration increased cell death up to 90% after 48 h treatment.

Thus, BML-210 can cause growth arrest in the transition through the cell cycle and induces cytotoxicity through the pathway of apoptosis.

### 2.2. BML-210 Inhibited HDAC Expression and Activity in NB4 Cells

**Figure 2:**
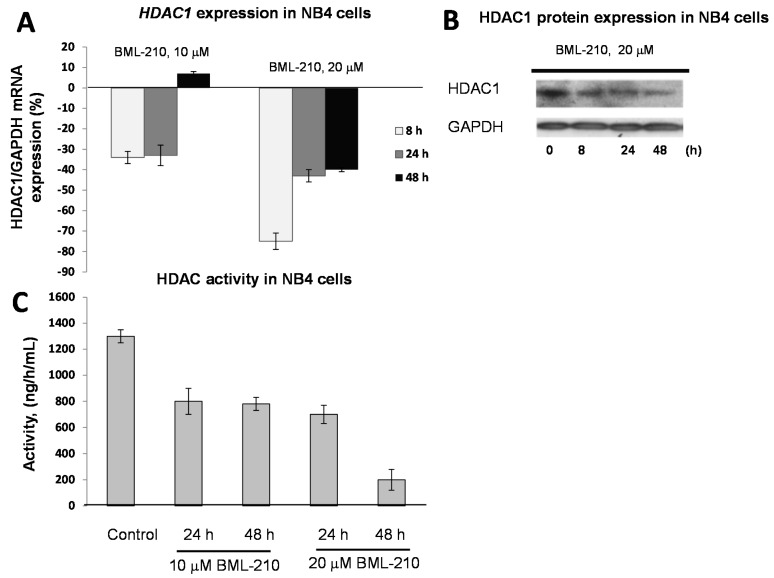
Expression of HDAC1 in response to BML-210 treatment. HDAC activity. (**A**) Expression levels of *HDAC1* were determined by RT-PCR analysis. Cells were exposed to 10 or 20 μM of BML-210 for two days. The results are presented as % from control cells (untreated); (**B**) HDAC1 protein expression. Cells were exposed to 20 μM of BML-210 for 2 days. Equal amounts of proteins from cell lysates were electrophoresed, and Western blot analysis was performed using antibodies against HDAC1 and GAPDH (as a loading control); (**C**) HDAC activity. Cells were exposed to 10 or 20 μM of BML-210 for two days. Activity of HDACs was measured using EpiQuik™ HDAC Activity/Inhibition Assay Kit. Results are given as mean ± S.E.M. (*n* = 3).

To determine effects of BML-210, as HDAC inhibitor, on HDAC expression level in NB4 cells, we performed gene (HDAC1, 2 and 3) expression experiments and Western blotting of HDAC1 protein (Figure 2).

BML-210 at 10 μM dose inhibited gene expression up to 36% after 48 h of treatment (Figure 2A). The 20 μM concentration of BML-210 inhibited HDAC expression up to 74% at 8 h point and then inhibition level reached almost the same point as after treatment with 10 μM BML-210 (40%) (Figure 2A). The changes in expression of HDAC 2 and HDAC 3 were very low and not significant (data not shown). The HDAC1 protein expression level was lowest after 48 h of treatment with 20 μM of BML-210 (Figure 2B).

For HDAC activity experiments, NB4 cells were treated with 10 and 20 μM BML-210 for 24, 48 h. Absorbance at 450 nm was estimated with spectrophotometer and HDAC activity was calculated using formula depicted in methods. It was noticed that after all treatments activity of HDAC decreases (Figure 2C). In NB4 cell line the maximum decrease (85%) of the activity was noticed after 48 h after 20 μM BML-210 treatment.

### 2.3. Proteomic Analysis of Protein Changes during Apoptosis of NB4 Cells after Treatment with BML-210

**Figure 3:**
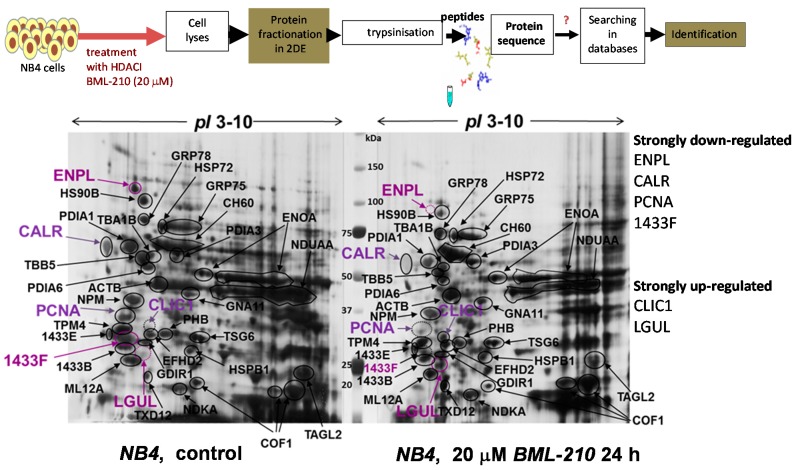
Proteomic analysis of proteins after BML-210 treatment. Proteins from untreated NB4 cells and cells treated with 20 μM BML-210 for 24 h fractionated in 2-DE system and visualized by Coomassie staining. Identified proteins shown, are listed in Table 1. Migration of the molecular size marker proteins is indicated in the center (kDa).

Proliferating (control) and induced to apoptosis with 20 µM BML-210 for 24 h NB4 cells were lysed and soluble cell proteins were resolved by 2-DE using pH range 3–10 and visualized by Coomassie staining (Figure 3). A proteomic approach was employed for identification of these proteins. Proteins were cut out and prepared for mass spectrometry analysis [12,13]. The software packages, MS-Fit and Mascot, were used to identify protein spots. In Table 1 summarized results of identified proteins are presented. Table S1 presents peptide sequences of identified proteins. Some protein spots could not be identified due to low protein concentration. By using mass spectrometry analysis, 35 proteins were identified.

**Table 1:** Identified proteins in NB4 cells after treatment with histone deacetylase inhibitor BML-210.

Name	Accession Number	*M*_W_ (Da)/pI	Name	Accession Number	*M*_W_ (Da)/pI	Name	Accession Number	*M*_W_ (Da)/pI
1433B_HUMAN 14-3-3 protein β/α	P31946	28,179/4.76	GRP78_HUMAN 78 kDa glucose-regulated protein	P11021	72,402/5.07	TAGL2_HUMAN Transgelin-2	P37802	22,548/8.41
1433E_HUMAN 14-3-3 protein eta	P62258	29,326/4.63	HS90B_HUMAN Heat shock 84 kDa (HSP84)	P08238	83,264/4.97	TBA1B_HUMAN Tubulin α-1B chain	P68363	50,804/4.94
1433F_HUMAN 14-3-3 protein eta	Q04917	28,372/4.76	HSP72_HUMAN Heat shock-related 70 kDa protein 2	P54652	70,022/5.6	TBB5_HUMAN Tubulin β chain	P07437	50,095/4.78
ACTB_HUMAN Actin, cytoplasmic 1	P60709	42,052/5.29	HSPB1_HUMAN Heat shock protein β-1	P04792	22,783/6.0	TSG6_HUMAN Tumor necrosis factor-inducible gene 6 protein	P98066	31,204/6.3
CALR_HUMAN Calreticulin	P27797	48,142/4.29	LGUL_HUMAN Lactoylglutathione lyase	Q04760	20,992/5.12	TPM4_HUMAN Tropomyosin α-4	P67936	28,619/4.67
CH60_HUMAN 60 kDa heat shock protein, mitochondrial	P10809	61,187/5.70	ML12A_HUMAN Myosin regulatory light chain 12A	P19105	19,839/4.67	TXD12_HUMAN Thioredoxin domain-containing protein 12	O95881	19,365/5.24
CLIC1_HUMAN Chloride intracellular channel protein 1	O00299	27,248/5.09	NDKA_HUMAN Nucleoside diphosphate kinase A	P15531	17,149/5.8	TAGL2_HUMAN Transgelin-2	P37802	22,548/8.41
COF1_HUMAN Cofilin-1	P23528	18,503/8.2	NDUAA_HUMAN NADH dehydrogenase 1α subcomplex subunit 10	O95299	41,067/8.67	TBA1B_HUMAN Tubulin α-1B chain	P68363	50,804/4.94
EFHD2_HUMAN EF-hand domain-containing protein D2	Q96C19	26,795/5.15	NPM_HUMAN Nucleophosmin	P06748	32,726/4.64			
ENOA_HUMAN α-Enolase	P06733	47,481/7.01	PCNA_HUMAN Proliferating cell nuclear antigen	P12004	29,092/4.57			
ENPL_HUMAN Endoplasmin (Heat shock protein 90 kDa β member 1)	P14625	92,469/4.76	PDIA1_HUMAN Protein disulfide-isomerase	P07237	57,480/4.76			
GDIR1_HUMAN ρ GDP-dissociation inhibitor 1	P52565	23,250/5.02	PDIA3_HUMAN Protein disulfide-isomerase A3	P30101	57,146/5.98			
GNA11_HUMAN Guanine nucleotide-binding protein subunit α-11	P29992	42,124/5.5	PDIA6_HUMAN Protein disulfide-isomerase A6	Q15084	48,490/4.95			

Some of the proteins are important for cell growth and/or homeostasis, such as α/β-tubulin, β-actin, cofilin-1, tropomyosin, myosin regulatory light chain 12A and gelsolin. Others are involved in protein metabolism (disulphide isomerase, α-enolase) and in protein folding (the heat shock proteins like endoplasmin, HSP90B, GRP75, GRP78 and CH60). Yet another group (PCNA, nucleophosmin, guanine nucleotide-binding protein subunit α-11, 14-3-3 protein, chloride intracellular channel protein 1, prohibitin and nucleoside diphosphate kinase A) comprised proteins responsible for signal transduction, cell differentiation, apoptosis and cell communication processes.

We determined that after 20 μM BML-210 treatment some proteins were down-regulated (endoplasmin, ENPL; heat shock 84 kDa, HSP90B; calreticulin, CALR; 14-3-3 protein eta, 1433F; proliferating cell nuclear antigen, PCNA) and few proteins up-regulated (chloride intracellular channel protein 1, CLIC1; lactoylglutathione lyase, LGUL; thioredoxin domain-containing protein 12, TXD12).

### 2.4. Changes of PCNA, ENPL, CALR, 1433F, CLIC1 and LGUL during Apoptosis of NB4 Cells after Treatment with BML-210

We performed RT-PCR experiments to evaluate changes at the gene level. We chose genes, for which expression level of products (proteins) were changed after 20 µM BLM-210 treatment: endoplasmin, ENPL; calreticulin, CALR; 14-3-3 protein eta, 1433F; proliferating cell nuclear antigen, PCNA; chloride intracellular channel protein 1, CLIC1; lactoylglutathione lyase, LGUL.

**Figure 4:**
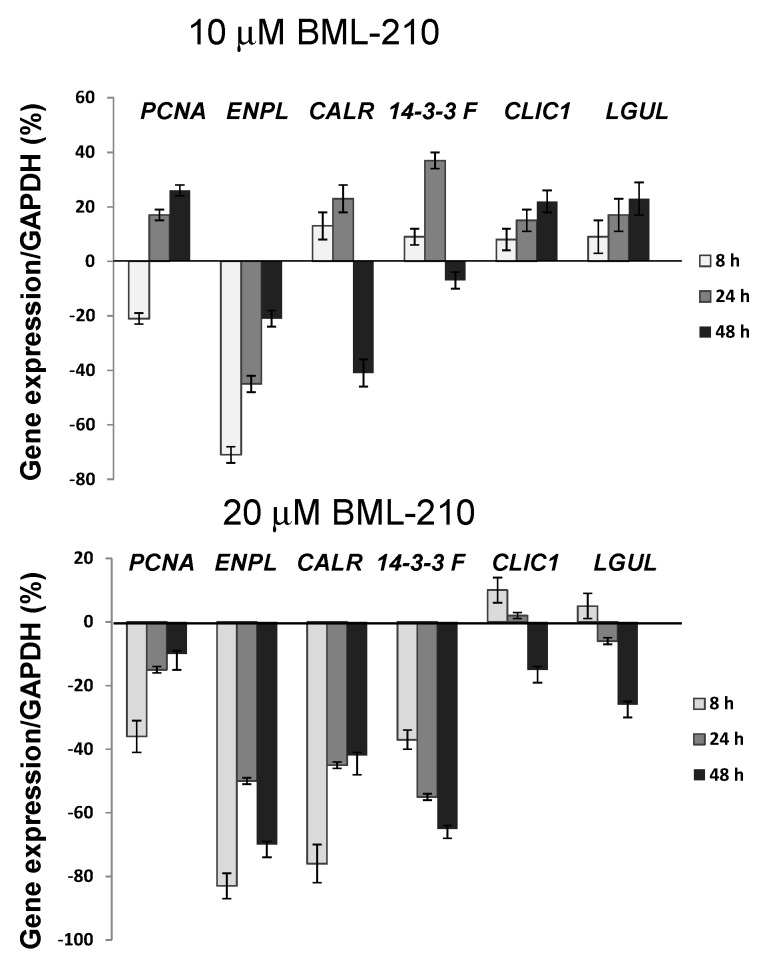
Changes of *PCNA*, *ENPL*, *CALR*, *1433F*, *CLIC1* and *LGUL* during treatment with BML-210. Cells were exposed to 10 or 20 µM of BML-210 for two days. The expression levels of *PCNA*, *ENPL*, *CALR*, *1433F*, *CLIC1* and *LGUL* were determined by RT-PCR analysis. The results presented as % from control cells (untreated). Results are given as mean ± S.E.M. (*n* = 3).

We determined that *PCNA* gene expression was inhibited after 8 h treatment with 10 µM BML-210 (20%) and up to 40% with 20 µM BML-210 (Figure 4). The *ENPL* gene expression was inhibited up to 70%–80% both with 10 and 20 µM BML-210. *CALR* the biggest changes were after 20 µM BML-210 (up to 80%) at the 8 h point. Expression of *1433F* was inhibited after 20 µM BML-210 treatment, but 10 µM BML-210 treatment increased *1433F* gene expression during 24 h of treatment. *CLIC1* and *LGUL* was up-regulated during 10 µM BML-210 treatment, and after 20 µM BML-210 treatment, gene expression was up-regulated at 8–24 h points, and then down-regulated.

We observed correlation between protein expression (after 24 h) and gene expression (after 8–24 h) of PCNA, ENPL, CALR, 1433F, CLIC1 and LGUL after 20 μM BML-210 treatment.

## 3. Discussion

The present study was designed to investigate how HDACI BML-210 affects NB4 cell growth, viability, apoptosis as well as the expression of HDACs and its activity during these cellular processes. Also, we wanted to identify any new proteins that might be involved in proliferation and apoptosis processes affected by BML-210 treatment. For this purpose we used proteomic analysis.

NB4 cells were treated with 10 or 20 μM BML-210 for 48 h and cell growth, viability, cell cycle and apoptosis were assessed. We showed that after all treatments cell growth and viability was negatively affected in a dose- and time-dependent manner. Cell viability was mostly affected with 20 µM BML-210, especially at 48 h point of treatment. In the previous studies we demonstrated that HDACIs, such as FK228, BML-210, phenyl butyrate, and vitamin B3, in different combinations with ATRA, act as KG1 cell growth inhibitors [14]. Similarly, a combination of ATRA and BML-210 leads to cell growth inhibition with subsequent apoptosis in a treatment time-dependent manner in cervical carcinoma cells (HeLa) [15]. Such effects are promising for the efficient differentiation therapy.

HDACIs influence gene transcription through deacetylation of histones. In fact, acetylation works together with other post-translational modifications, and blocking deacetylation might have very different outcomes depending on the previous chromatin state. Also HDACI effects on DNA replication and repair, which can influence modulation of the cellular response to HDACI [16]. HDACI induce, to a variable extent, growth arrest, differentiation or apoptosis *in vitro* and *in vivo* [17,18]. In some cases, growth arrest is induced at low doses, and apoptosis is induced at higher doses; in other cases, growth arrest precedes apoptosis. The ability of HDACI to selectively induce tumor cells to undergo apoptosis is important for the therapeutic efficacy observed in pre-clinical models. *In vitro* low dose concentrations of HDACI can sensitize malignant cells to other drugs (retinoids, EGFR inhibitors, cell cycle inhibitors and many others) that can induce apoptosis.

Further, we investigated the expression of HDAC1, HDAC2 and HDAC3. HDACs are currently divided into four classes based on phylogenetic and functional criteria [19]. In our research HDACs from I and II classes were analyzed. Class I HDACs are structurally similar to yeast transcription factor, Rpd-3, and typically associate with multiprotein repressor complexes containing sin3, Co-REST, Mi2/NuRD, *N*-COR/SMRT, and EST1B [20,21,22]. HDACs 1, 2, and 3 are localized in the nucleus, and target multiple substrates including p53, myo-D, STAT-3, E2F1, Rel-A, and YY1 [20,21,22]. HDAC 4 belongs to class IIa together with HDACs 5, 7, 9. Proteins of this class contain large N-terminal domains that regulate DNA binding and interact in a phosphorylation-dependent manner with 14-3-3 proteins, which mediate movement of these HDACs between cytoplasm and nucleus in response to mitogenic signals [7].

NB4 cells were treated with BML-210 for 8, 24, 48 h. We determined only HDAC1 gene and protein expression changes after treatments with BML-210, especially after 20 μM dose treatment; we observed no changes in the expression of HDAC2 or HDAC3 level. Changes in HDAC expression during treatment with the HDAC inhibitor BML-210 can be caused by epigenetic changes, when using an inhibitor, deacetylation of histones decreases and genes those products control the expression of HDACs are activated. Krämer and colleagues showed that HDAC2 undergoes turnover by proteasomal degradation and this was induced by HDAC inhibitor Valproic acid (VPA) [23]. However, other HDAC inhibitors, such as trichostatin A (TSA) inhibits the catalytic activity of class I HDACs, but does not induce proteasomal degradation of HDAC2. In our study we did not observe a decrease in HDAC2 protein expression. On the other hand, we observed time- and dose-dependent HDAC activity inhibition by BML-210. The maximum decrease (85%) of the activity was noticed after 48 h with 20 μM BML-210 treatment. Therefore, we could suggest that BML-210 acts in a similar way as TSA, by selectively inhibiting the catalytic activity of HDACs class I, but not inducing the proteasomal degradation of HDAC2. Wagner and coauthors [24] explore the mechanisms connecting HDAC2 and p53 *in vitro* and *in vivo*. They summarize recent findings on HDAC2 overexpression in solid and hematopoietic cancers and suggest that HDAC2 regulates the tumor suppressor p53 by deacetylation and the maintenance of genomic stability. In our case, BML-210 acts as inhibitor of catalytic activity of HDACs, affects the acetylation status of both histone and non-histone proteins and modulates epigenetic changes in human promyelocytic leukemia NB4 cells inducing cell differentiation and apoptosis.

The processes in which HDACs, including partners, participate are rather complex and HDAC expression and activity regulation by various chemical agents could support potential therapeutic use in correcting the transcriptional deregulation of genes involved in cell cycle regulation and apoptosis of leukemic as well as other cancer cells.

To investigate what kind of proteins can be activated or inhibited after BML-210 treatment, we performed proteomic analysis. We used 20 µM concentration of BML-210. At this concentration we observed the strongest HDAC1 expression inhibition and HDAC activity inhibition. We identified 35 proteins. Some of them are important for cell growth and/or homeostasis, such as α/β-tubulin, β-actin, cofilin-1, tropomyosin, myosin regulatory light chain 12A and gelsolin. Others are involved in protein metabolism (disulphide isomerase, α-enolase) and in protein folding (the heat shock proteins like endoplasmin, HSP90B, GRP75, GRP78 and CH60). Yet another group (PCNA, nucleophosmin, guanine nucleotide-binding protein subunit α-11, 14-3-3 protein, chloride intracellular channel protein 1, prohibitin and nucleoside diphosphate kinase A) comprised proteins responsible for signal transduction, cell differentiation, apoptosis and cell communication processes. We determined that some of the identified proteins changed expression after treatment with BML-210. We prepared RT-PCR analysis of the genes whose products’ expression level were changed after 20 µM BLM-210 treatment: endoplasmin, ENPL; calreticulin, CALR; 14-3-3 protein eta, 1433F; proliferating cell nuclear antigen, PCNA; chloride intracellular channel protein 1, CLIC1; lactoylglutathione lyase, LGUL. Our results correlated with the proteomic data. We determined that after BML-210 (both 10 and 20 µM) treatment, endoplasmin, calreticulin, 14-3-3 protein eta, and proliferating cell nuclear antigen (PCNA) were down regulated. The products of these genes are important for the processes of cell proliferation, differentiation and apoptosis. The gene expression chloride intracellular channel protein 1, CLIC1; lactoylglutathione lyase, LGUL decreased only after 24 h treatment with 20 µM BML-210. It is known that the chloride channels proteins regulate fundamental cellular processes including stabilization of cell membrane and the LGUL has been suggested to play a role in regulating cell growth. Accordingly, it is likely that the decrease of the expression of these genes (CLIC1 and LGUL) is associated with cell apoptotic processes.

It was shown that HDACI can cause hyper-acetylation of HSP90 and its inactivation, leading to the degradation of proteins that require the chaperone function of HSP90 (including some oncoproteins) [25]. It has furthermore been shown that HSP90 inhibition correlated with growth arrest followed by differentiation and apoptosis [26,27]. Calreticulin function was studied by Sheng and co-workers [28]. They observed that high expression of calreticulin was positively associated with tumor stage and metastasis and that calreticulin regulated cell proliferation, migration and invasion of pancreatic cancer cells in a MEK/ERK pathway dependent manner. 14-3-3 proteins play important roles in a wide range of vital regulatory processes, including signal transduction, apoptosis, cell cycle progression and DNA replication. Grebenova and co-authors [29] showed that leukemic cell treatment by suberoylanilide hydroxamic acid (SAHA) induced cofilin phosphorylation, an increase in vimentin and paxillin expression and a decrease in stathmin expression. They also demonstrated that cofilin interacts with 14-3-3 epsilon.

It is known that PCNA is an essential factor for DNA replication and repair. We determined that PCNA is down-regulated in NB4 cells, which undergo apoptosis after BML-210 treatment.

We identified another group of proteins to be up-regulated after BML-210 treatment: chloride intracellular channel protein 1 and lactoylglutathione lyase. It was proposed that CLIC1 can be a novel potential prognostic factor, for which up-regulation is correlated with cancer development and metastasis [30]. Lactoylglutathione lyase is implicated in the progression of various human malignant diseases. It was shown by Wang and co-workers that overexpression and nuclear translocation of GLO1 might be associated with tumor progression in murine fibrosarcoma [31].

## 4. Methods

### 4.1. Chemicals

BML-210 was obtained from Biomol Research Laboratories/ Enzo Life Sciences, Inc (Farmingdale, New York, NY, USA). ATRA was purchased from Sigma Chemical Co. (St. Louis, MO, USA). The stock solution of BML-210 (5 mM in DMSO) was stored in −20 °C.

### 4.2. Cell Culture

The human acute leukemia cell line NB4 was maintained in RPMI-1640 medium supplemented with 10% fetal bovine serum, 100 U/mL penicillin and 100 μg/mL streptomycin (Gibco, Grand Island, NY, USA). Cells were grown at 37 °C in a humidified 5% CO_2_ atmosphere and used for assays during exponential phase of growth.

### 4.3. Assessment of Cell Proliferation, Apoptosis and Cell Cycle Distribution

Cell proliferation was evaluated by the trypan blue exclusion test. Viable and dead (blue colored) cell number was determined by counting in a hemocytometer. The growth inhibition was calculated from: [(*Cx* − *C*o) − (*Tx* − *T*o)/(*Cx* − *C*o)] × 100, where *C*o, *Cx*, *T*o and *Tx* represent the total number of cells/mL in untreated (C) and treated (T) cultures at days 0 and *x* (2 or 3), respectively. At least 400 cells were scored for each determination.

Apoptosis were monitored by quantification of cellular DNA content after staining with PI (Propidium iodide). Control and treated cells were collected by centrifugation, suspended in PBS and fixed in ice-cold 70% ethanol (ratio 1:10) for 24 h at −20 °C. After centrifugation at 500× *g* for 5 min, cells were suspended in PBS containing PI (50 μg/mL) and RNAse (0.2 mg/mL) and incubated at room temperature for 30 min. The tubes were then taken at 4 °C in dark until flow cytometric analysis in FACSCalibur (Becton-Dickinson, San Jose, CA, USA). Apoptotic cells were quantified on PI histogram as a hypodiploid peak (SubG1) and the data was registered on a logarithmic scale.

### 4.4. HDAC Activity Analysis

Proteins were isolated using ProteoJET™ Mammalian Cell Lysis Reagent (Fermentas, Vilnius, Lithuania) according to the manufacturer's instructions.

HDAC activity analysis was determined using EpiQuik™ HDAC Activity/Inhibition Assay Kit (Epigentek Group Inc., Brooklyn, NY, USA) according to the manufacturer’s instructions. Absorbance was measured using Tecan-Control, Infinite 200 microplate reader at 450 nm. Activity (ng/h/mL) was calculated using formula:

(1) $$Activity(ng/h/mL)\;=\frac{OD(control-blank)-OD(sample-blank)}{tg\alpha \times t}\times S$$

Here: *OD*: optical density; *Blank*: well only with buffer; *Control*: well in which enzyme is not added, there is only acetylated substrate; *tgα*: slope in OD dependence on HDAC concentration curve; *t*: reaction time; *S*: sample dilution.

### 4.5. Isolation of Proteins for Western Blot Analysis

Cells were harvested by centrifugation at 250× *g* for 7 min and washed twice in PBS (pH 7.5). 1/10 volume of benzonase was added and sediments were kept on ice for 10 min, later 10 volumes of lysis solution was added (150 mM NaCl, 10 mM Tris/HCl (pH 7.5), 5 mM EDTA, 1% Nonidet P-40, 1 mM PMSF, 5 mM NaF, 1 mM Na_3_VO_4_ and 1× protease inhibitor cocktail (Roche, Basel, Switzerland)). The lysates were homogenized and centrifuged at 14,000× *g* for 20 min. The supernatant corresponding total protein was used for electrophoresis directly or stored in −20 °C.

Proteins were resolved by SDS/PAGE electrophoresis, equal amounts of proteins from cell lysates were electrophoresed. For this technique, we used a 10% polyacrylamide gradient gel in Tris-glycine electrophoresis buffer. After SDS electrophoresis, proteins were transferred to Immobilon™ PVDF transfer membranes and blocked by incubating overnight at 4 °C with 5% BSA dissolved in PBS containing 0.18% Tween-20. The membranes were incubated for 1 h with antibodies against HDAC1 (Cell Signaling technology, Danvers, MA, USA), GAPDH (Abcam, Cambridge, UK) at a concentration of 1 µg/mL in PBS. The membranes were subsequently washed with PBS-Tween-20 and then incubated with horseradish peroxidase-conjugated secondary antibody (DAKO, A/S, Copenhagen, Denmark) for 1 h at room temperature. Thereafter, the filters were washed as described and immunoreactive bands detected by enhanced chemiluminescence using ECL™ Western blotting detection reagents (GE Healthcare Amersham, Buckinghamshire, UK), according to the instructions of the manufacturer.

### 4.6. 2DE Electrophoresis

For 2DE, the total soluble fractions were actively solubilized in 80 µL of IEF buffer (8 M urea, 2 M thiourea, 4% CHAPS, 1% DTT (dithiothreitol), 0.002% Bromphenol blue, 0.8% Pharmalyte 3-10) and in 120 µL of rehydration buffer (8 M urea, 2 M thiourea, 2% CHAPS, 0.2% DTT, 0.002% bromphenol blue, 0.5% pharmalyte, pH range 4–7), and then Immobiline DryStrips strips, pH 3–10 (GE Healthcare, Milwaukee, WI, USA), were hydrated with the rehydration solution in a strip holder for 16 h at room temperature. The first dimension of isoelectric focusing was performed with a 2DE Multiphor unit (GE Healthcare) by steps of increased voltage up to ~30 kVh.

Focusing Immobiline DryStrips were incubated for 15 min at room temperature in electrophoresis buffer (50 mM Tris-HCl, pH 8.8, 6 M urea, 30% (*w*/*v*) glycerol, 2% (*w*/*v*) SDS) with 1% (*w*/*v*) DTT, followed by 15 min in electrophoresis buffer with 2% (*w*/*v*) iodoacetamide.

The second dimension was performed in a gradient gel (8%–18% ExcelGel SDS polyacrylamide). 2DE gels were Coomassie-stained. Spots were selected by visual inspection and gel slices were excised by scalpel.

### 4.7. Mass Spectrometry Analysis

The gel areas of interest were cut out and subjected to in-gel tryptic digestion according to Shevchenko *et al.* [12,13]. For MALDI-TOF-MS analysis, samples were desalted and purified using C18zipTips (EMD Millipore, Billerica, MA, USA) following the manufacturer's instructions (EMD Millipore). After that, 1 µL of each sample was mixed with 1 µL α-cyano-4-hydroxicinnamic acid matrix (in 70% acetonitrile with 0.3% (*v*/*v*) trifluoroacetic acid) and spotted on the target plate. Samples were analyzed with a MALDI-TOF-MS using a Voyager-DE™ Pro (Applied Biosystems, Framingham, MA, USA). Positive ionization, acceleration voltage 20 kV, grid voltage 75%, guide wire 0.02 and the extraction delay time 200 ns were used to collect spectra in the mass range of 700–4000 Da. Reflector mass spectra were acquired and calibrated either externally or internally, using trypsin autolysis peptides (*m*/*z* 842.5200, 1045.5642, 2211.1046). Data processing of the spectra was performed with Data Explorer™ Version 4.0 (Applied Biosystems). Mass spectrometry data were searched against the human protein database (a subset of proteins from the NCBI non-redundant protein database) using the MASCOT software search algorithms (Matrix Science Ltd., London, UK). Restrictions were passed on maximum missed cleavages by trypsin (up to 1), and cysteine modification by carbamidomethylation.

For detection of spots where protein expression level changed after BML-210 treatment (PCNA, ENPL, HSP90, CALR, 1433F, CLIC1, TXD12 and LGUL) we used another identification method: the peptides were chromatographically separated using Agilent 1100 HPLC system with the flow splitter and analyzed by electrospray ionization MS in positive ionization mode using the ion trap “HCTultra PTM Discovery System” (Bruker Daltonics, Bremen, Germany). A C18 reverse phase column (5 µm; 0.3 × 150 mm) and a flow rate of 7 µL/min were used. A gradient of 0.1% formic acid in water (A) and 0.1% formic acid in acetonitrile (B) was distributed as follow: 0%–5% B in first 30 min; 5%–15% B in 30–70 min; 15%–30% B in 70–100 min; 30%–40% B in 100–120 min; 40%–70% B in 120–150 min and 100% B in 150–170 min. The automated online tandem MS analyses were performed using collision induced dissociation of peptide ions.

### 4.8. Quantitative Real-Time PCR (RT-qPCR)

Total RNA was extracted by TRIzol (Thermo Fisher Scientific, Waltham, MA, USA), as recommended by the manufacturer, and then reverse transcribed into cDNA using Maxima First Strand cDNA Synthesis Kit (Thermo Scientific, Vilnius, Lithuania) or TaqMan^®^ MicroRNA Reverse Transcription Kit (Life Technologies/Thermo Fisher Scientific, Waltham, MA, USA). RT-qPCR was performed with Maxima^®^ SYBR Green qPCR Master Mix (Thermo Scientific, Vilnius, Lithuania) or TaqMan^®^ Universal Master Mix II with TaqMan^®^ MicroRNA Assay (Life Technologies, Waltham, MA, USA) on the Rotor-Gene 6000 system (Corbett Life Science, Sydney, Australia). The primers (5ʹ–3ʹ orientation) used were as follows (Table 2):

**Table 2:** Primer sets used for RT-qPCR.

Gene	Primers	Annealing Temperature (°C)
*GAPDH*	F: GGAAGTCAGTTCAGACTCCAGCC	60
	R: AGGCCTTTTGACTGTAATCACACC	
*HDAC-1*	F: CAAGCTCCACATCAGTCCTTC	64
	R: TGCGGCAGCATTCTAAGGTT	
*HDAC-2*	F: AGTCAAGGAGGCGGCAAAA	64
	R: TGCGGCAGCATTCTAAGGTT	
*HDAC-3*	F: CCGAAATGTTGCCCGCTGCTG	64
	R: AGGTGCATGGTTCAGCATCTT	
*CALR*	F: AGTTCCGGCAAGTTCTACGG	58
	R: ACAGAGCATAAAAGCGTGCAT	
*CLIC1*	F: ACCGCAGGTCGAATTGTTC	58
	R: ACGGTGGTAACATTGAAGGTG	
*ENPL*	F: GCTGACGATGAAGTTGATGTGG	58
	R: CATCCGTCCTTGATCCTTCTCTA	
*LGUL*	F: TGACCATTGTGCTCTTGGCT	58
	R: ATGTGAATCATGGCGGGGAA	
*PCNA*	F: GCGTGAACCTCACCAGTATGT	58
	R: TCTTCGGCCCTTAGTGTAATGAT	
*1433F*	F1: TGGCTGATGGAAACGAAAAGAA	58
	R1: CCTCTGCTAAGTAGCGGTAGT	

The amount of mRNA was normalized to GAPDH. The relative gene expression was calculated by a comparative threshold cycle Δ-Δ *C*_t_ method.

### 4.9. Statistical Analysis

For statistical analysis, Student’s *t* test was used to determine the significance of differences between groups for paired samples. Data from at least 3 experiments are shown as typical result or as mean ± S.E.M.

## 5. Conclusions

In general, our data suggest, that treatment of cells with BML-210 is correlated with inhibition of HDAC activity and changes in HDAC1 expression. These results indicate that, HDACI BML-210 can affect proteins that are important for proliferation and apoptosis of leukemic cells, and therefore could be important to find new targets in cancer treatment.

## Supplementary Materials

Supplementary materials can be found at: http://www.mdpi.com/1422-0067/16/08/18252/s1.

## Abbreviations

AML: Acute myeloid leukemia
APL: Acute promyelocytic leukemia
ATRA: All-*trans* retinoic acid
ECL: Enhanced chemiluminescence
GAPDH: glyceraldehide-3-phosphate dehydrogenase
HAT: Histone acetyltransferase
HDAC: Histone deacetylase
HDACI: Histone deacetylase inhibitor
NBT: Nitro blue tetrazolium
PBS: Phosphate buffered saline
PML: Promyelocytic leukemia gene
PMSF: Phenylmethylsulfonyl fluoride
RARα: Retinoic acid receptor α
