# Supplementary material for: The Histone Deacetylase Inhibitor BML-210 Influences Gene and Protein Expression in Human Promyelocytic Leukemia NB4 Cells via Epigenetic Reprogramming
Source: Int J Mol Sci. 2015 Jun 6;16(8):18252–69. doi: 10.3390/ijms160818252 (PMC4581243; doi:10.3390/ijms160818252)
Supplement: Supplementary file 1 [file ijms-16-18252-s001.pdf]

Supplementary Information

Table S1. Identified proteins and peptide sequences in NB4 cells after treatment with histone deacetylase inhibitor BML-210.

Name	Accession Number	M_W (Da)/pI	Sequence cov. (%)	Peptides		
1433B_HUMAN	P31946	28,179/4.76	30%	-.MTMDKSELVQK.A	R.YLSEVASGDNK.Q	K.VFYLKMK.G
14-3-3 protein β/α				K.AVTEQGHELSNEER.N	K.EMQPTHPIR.L	K.MKGDYFR.Y
				R.VISSIEQK.T	K.DSTLIMQLLR.D	
1433E_HUMAN	P62258	29,326/4.63	25%	K.VAGMDVELTVEER.N	K.AASDIAMTELPPTHPIR.L	R.YLAEFATGNDR.K
14-3-3 protein eta				K.LICCDILDVLDK.H	K.DSTLIMQLLR.D	
1433F_HUMAN	Q04917	28,372/4.76	61%	-.MGDREQLLQR.A	K.ELETVCNDVLSLLDKFLIK.N	R.NLLSVAYK.N
14-3-3 protein eta				R.EQLLQR.A	K.VFYLKMK.G	K.NVVGARR.S
				R.LAEQAER.Y	K.MKGDYYR.Y	R.VISSIEQK.T
				R.YDDMASAMK.A	R.YLAEVASGEK.K	K.TMADGNEK.K
				K.AVTELNEPLSNEDR.N	K.NSVVEASEAAYK.E	K.EAFEISK.E
					K.DSTLIMQLLR.D	K.EAFEISKEQMQPTHPIR.L
ACTB_HUMAN	P60709	42,052/5.29	46%	K.AGFAGDDAPR.A	R.EIVRDIK.E	K.EITALAPSTMK.I
Actin, cytoplasmic 1				R.AVFPSIVGRPR.H	K.SYELPDGQVITIGNER.F	K.IKIIAPPER.K
				R.HQGVMVGMGQK.D	K.CDVDIR.K	R.DLTDYLMK.I
				K.IWHHTFYNELR.V	R.VAPEEHPVLLTEAPLNPK.A	R.GYSFTTTAER.E
				K.DLYANTVLSGGTTMYPGIADR.M	K.RGILTLK.Y	R.MQKEITALAPSTMK.I
CALR_HUMAN	P27797	48,142/4.29	36%	.LKEEEEDK.K	K.GQTLVVQFTVK.H R	K.KVHVIFNYK.G
Calreticulin				K.VHVIFNYK.G	K.EQFLDGDGWTSR.W	K.DKGLQTSQDAR.F
				K.DKQDEEQR.L	K.HEQNIDCGGGYVK.L	
				K.FYGDEEKDK.G	K.IKDPDASKPEDWDER.A	

Table S1. *Cont.*

Name	Accession Number	M_W (Da)/pI	Sequence cov. (%)	Peptides		
CH60_HUMAN<br>60 kDa heat shock protein, mitochondrial	P10809	61,187/5.70	56 %	-.MLRLPTVFR.Q<br>R.LPTVFR.Q<br>R.QMRPVSR.V<br>R.AYAKDVK.F<br>R.TVIIEQSWGSPK.V<br>K.YKNIGAK.L<br>R.KPLVIIAEDVDGEALSTLVLNR.L<br>K.LVQDVANNTNEEAGDGTTTATVLAR.S<br>K.GANPVEIR.R<br>R.RGVMLAVDAVIAELK.K<br>K.EIGNIISDAMK.K<br>R.KGVITVK.D<br>K.TLNDELEIIEGMK.F	K.KISSIQSIVPALEIANAHR.K<br>K.ISSIQSIVPALEIANAHR.K<br>R.LKVGLQVVAVK.A<br>K.VGLQVVAVK.A<br>K.APGFGDNR.K<br>K.APGFGDNRK.N<br>K.VGEVIVTK.D<br>K.AQIEKR.I<br>R.GYISPYFINTSK.G<br>K.CEFQDAYVLLSEK.K<br>K.IPAMTIAK.N<br>K.NAGVEGSLIVEK.I<br>K.GIIDPTKVVR.T	K.RIQEIIEQLDVTTSEYEK.E<br>R.IQEIIEQLDVTTSEYEK.E<br>K.LNERLAK.L<br>K.LSDGVAVLK.V<br>K.DRVTDALNATR.A<br>R.VTDALNATR.A<br>R.AAVEEGIVLGGGCALLR.C<br>K.IGIEIIK.R<br>K.IGIEIIKR.T
CLIC1_HUMAN<br>Chloride intracellular channel protein 1	O00299	27,248/5.09	70%	KLCPGGQLPFLLYGTEVHTDTNKI<br>R.EEFASTCPDDEEIELAYEQVAK.A<br>K.LAALNPESNTAGLDIFAK.F<br>K.VLDNYLTSPLPEEVDETSAEDEGSQR.K	R.KFLDGNELTLADCNLLPK.L<br>K.LHIVQVVCK.K<br>R.GFTIPEFR.G<br>R.YLSNAYAR.E	K.IEEFLEAVLCPPR.Y<br>K.GVTFNVTTVDTK.R<br>K.NSNPALNDNLEK.G
COF1_HUMAN<br>Cofilin-1	P23528	18,503/8.2	74%	K.VFNDMKVR.K<br>K.SSTPEEVK.K<br>K.AVLFCLSEDK.K	K.MLPDKDCR.Y<br>R.YALYDATYETK.E<br>K.EDLVFIFWAPESAPLK.S<br>K.EILVGDVGQTVDDPYATFVK	K.MIYASSKDAIK.K<br>K.HELQANCYEEVK.D<br>K.LGGSAVISLEGKPL.-<br>K.KNIILEEGK.E
EFHD2_HUMAN<br>EF-hand domain-containing protein D2	Q96C19	26,795/5.15	35%	R.ADLNQGIGEPQSPSR.R<br>R.DGFIDLMELK.L<br>K.AAAGELQEDSGLCVLAR.L	K.VQAINVSSR.F<br>R.FEEEIKAEQEER.K	K.EVDEDFDSK.L<br>R.LSEIDVSSEGVK.G
ENOA_HUMAN<br>A-enolase	P06733	47,481/7.01	16%	R.GNPTVEVDLFTSK.G<br>R.AAVPSGASTGIYEALELR.D	K.VVIGMDVAASEFFR.S<br>R.YISPDQLADLYK.S	K.LAQANGWGVMVSHR.S

Table S1. *Cont.*

Name	Accession Number	M_W (Da)/pI	Sequence cov. (%)	Peptides		
ENPL_HUMAN	P14625	92,469/4.76	20%	R.EEEAIQLDGLNASQIR.E	R.GLFDEYGSK.K	K.SGTSEFLNK.M
Endoplasmin				K.FAFQAEVNR.M	K.GVVDSDDLPLNVSR.E	R.GTTITLVLK.E
(Heat shock protein				K.LIINSLYK.N	R.FQSSHHPTDITSLDQYVER.M	K.EEASDYLELDTIK.N
90 kDa β member 1)				R.ELISNASDALDK.I	K.AEASSPFVER.L	K.EVEEDEYK.A
				R.LSLNIDPDAK.V	K.DISTNYYASQK.K	R.SGYLLPDTK.A
GDIR1_HUMAN	P52565	23,250/5.02	9%	K.EGVEYR.I		
ρ GDP-dissociation				K.YIQHTYR.K		
inhibitor 1				R.FTDDDK.T		
GNA11_HUMAN	P29992	42,124/5.5	26%	R.GFTKLVYQNIFTAMQAMIR.A	K.KDLLEDK.I	K.DLLEDK.I
Guanine nucleotide-				R.TIITYPWFQNSSVILFLNK.K	R.MVDVGGQR.S	K.ANALLIR.E
binding protein				-.MTLESMMACCLSDEVKESK.R	K.YYLTDVDR.I	
subunit α-11						
GRP75_HUMAN	P38646	73,920/5.87	25%	R.TTPSVVAFTADGER.L	K.SDIGEVILVGGMTR.M	K.YAEEDR.R
Stress-70 protein,				R.LVGMPAKR.Q	K.VQQTVQDLFGR.A	K.EEISKMR.E
mitochondrial				K.NAVITVPAYFNDSQR.Q	K.LLGQFTLIGIPPAPR.G	R.ELLARK.D
				K.DAGQISGLNVLR.V	R.AQFEGIVTDLIR.R R.VREAAEK.A	K.KMASER.E R.NTTIPTK.K
				K.STNGDTFLGGEDFDQALLR.H	R.LVGMPAK.R	
				R.ETGVDLTKDNMALQR.V		
GRP78_HUMAN	P11021	72,402/5.07	36%	R.ITPSYVAFTPEGER.L	K.FEELNMDLFR.S	R.IPKIQQLVK.E
78 kDa				R.TWNDPSVQQDIK.F	K.VLEDSDLKK.S	K.EFFNGKEPSR.G
glucose-regulated				K.TKPYIQVDIGGGQTK.T	K.KSDIDEIVLVGGSTR.I	K.VYEGERPLTK.D
protein				K.KVTHAVVTVPAYFNDAQR.Q	K.SDIDEIVLVGGSTR.I	K.ITITNDQNR.L
				K.VTHAVVTVPAYFNDAQR.Q	K.DNHLLGTFDLTGIPPAPR.G	R.LTPEEIER.M
				K.DAGTIAGLNVMR.I	K.IEWLESHQDADIEDFK.A	R.VEIIANDQGNR.I
				R.IINEPTAAAIAYGLDKR.E	R.ALSSQHQAR.I	
				R.IEIESFYEGEDFSETLTR.A	R.AKFEELNMDLFR.S	

Table S1. *Cont.*

Name	Accession Number	M_W (Da)/pI	Sequence cov. (%)	Peptides		
HS90B_HUMAN Heat shock 84 kDa (HSP84)	P08238	83,264/4.97	32%	K.HNDDEQYAWESSAGGSFTVR.A	K.YIDQEELNK.T	K.EIFLR.E
				R.ADHGEPIGR.G	R.NPDDITQEEYGEFYK.S	R.ELISNASDALDK.I
				K.EDQTEYLEER.R	K.SLTNDWEDHLAVK.H	R.YESLTDPSK.L
				K.FENLCK.L	R.ALLFIPR.R	K.IDIIPNPQER.T
				K.LGLGIDEDEVAAEEPNAAVPDEIPPLEDEDASR.M	R.GVVDSEDLPLNISR.E	K.ADLINNLGTIAK.S
				K.EQVANSAFVER.V	K.LGIHEDSTNR.R	K.SLVSVTK.E
					K.EGLELPEDEEEK.K	K.SIYYITGESK.E
HSP72_HUMAN Heat shock-related 70 kDa protein 2	P54652	70,022/5.6	7%	R.ARFEELNADLFR.G	R.FEELNADLFR.G	
				K.STAGDTHLGGEDFDNR.M	R.TTPSYVAFTDTER.L	
HSPB1_HUMAN Heat shock protein β1	P04792	22,783/ 6.0	40%	R.VPFSLLR.G	R.LFDQAFGLPR.L	R.DWYPHSR.L
				K.DGVVEITGK.H	R.VSLDVNHFAPDELTVK.T	R.GPSWDPFR.D
				R.QLSSGVSEIR.H	K.LATQSNEITIPVTFESR.A	
LGUL_HUMAN Lactoylglutathione lyase	Q04760	20,992/5.12	18%	R.VLGMTLIQK.C	K.RFEELGVK.F	
				R.GFGHIGIAVPDVYSACK.R		
ML12A_HUMAN Myosin regulatory light chain 12A	P19105	19,839/4.67	29%	K.EAFNMIDQNR.D	K.GNFNYIEFTR.I R.FTDEEVDELYR.E	
				R.LTGDAFR.KACFDEEATGTIQEDYLR.E		
NDKA_HUMAN Nucleoside diphosphate kinase A	P15531	17,149/ 5.8	20%	R.NIIHGSDSVESAEK.E	R.TFIAIKPDGVQR.G	
				R.VMLGETNPADSKPGTIR.G		
NDUAA_HUMAN NADH dehydrogenase 1 α subcomplex subunit 10	O95299	41,067/8.67	28%	K.LAATSASARVVAAGAQR.V	R.GIHSSVQCKLR.Y	R.VLHQFRELPGR.K
				R.SIFSDFVFLEAMYNQGFIR.K	R.SRVITVDGNICTGK.G	R.LLKLAATSASAR.V
				R.TLYHLRLLVQDK.F	K.FYDDPRSNDGNSYR.L	

Table S1. *Cont.*

Name	Accession Number	M_W (Da)/pI	Sequence cov. (%)	Peptides		
NPM_HUMAN Nucleophosmin	P06748	32,726/4.64	62%	K.ADKDYHFK.V	K.DSKPSSTPR.S	R.SKGQESFK.K
				K.DYHFK.V	K.DSKPSSTPRSK.G	K.GQESFKK.Q
				K.VDNDENEHQLSLR.T	R.MTDQEAIQDLWQWR.K	K.QEKTPK.T
				R.TVSLGAGAK.D	K.RSAPGGGSK.V	K.GPSSVEDIK.A
				K.DELHIVEAEAMNYEGSPIK.V	K.SIRDTPAK.N	K.AKMQASIEK.G
				K.VTLATLK.M	K.NAQKSNQNGK.D	K.MQASIEK.G
				K.MSVQPTVSLGGFEITPPVVLR.L	K.SNQNGKDSKPSSTPR.S	K.GGSLPKVEAK.F
				K.LLSISGK.R		K.FINYVK.N
PCNA_HUMAN Proliferating cell nuclear antigen	P12004	29,092/4.57	40%	R.NLAMGVNLTSMSK.I	R.DLSHIGDAVVISCAK.D	R.YLNFFTK.A
				R.AEDNADTLALVFEAPNQEK.V	R.LVQGSILK.K	K.YYLAPK.I K.MPSGEFAR.I
				K.LMDLDVEQLGIPEQEYSCVVK.M	R.SEGFDTYR.C	
PDIA1_HUMAN Protein disulfide-isomerase	P07237	57,480/4.76	54%	R.KSNFAEALAAHK.Y	K.SVSDYDGKLSNFK.T	K.IFGGEIK.T
				K.SNFAEALAAHK.Y	K.TAAESFK.G	K.THILLFLPK.S
				K.ALAPEYAK.A	K.ILFIFIDSDHTDNQR.I	K.SVSDYDGK.L
				K.LKAEGSEIR.L	R.LITLEEEMTK.Y	R.ILEFFGLK.K
				K.AEGSEIR.L	K.YKPESEELTAER.I	K.KEECPAVR.L
				K.VDATEESDLAQQYGVR.G	R.ITEFCHR.F	K.EECPAVR.L
				K.FFRNGDTASPK.E	K.NFEDVAFDEK.K	K.KFDEGR.N
				R.NGDTASPK.E	K.QLAPIWDK.L	R.NNFEGEVTK.E
				R.EADDIVNWLK.K	K.LGETYK.D	K.ENLLDFIK.H
				K.YQLDKDGVVLFK.K	K.LGETYKDHENIVIAK.M	K.FFPASADR.T
				K.HNQLPLVIEFTEQTAPK.I	K.VHSFPTLK.F	K.DGVVLFK.K
						R.TVIDYNGER.T

Table S1. *Cont.*

Name	Accession Number	M_W (Da)/pI	Sequence cov. (%)	Peptides		
PDIA3_HUMAN	P30101	57,146/5.98	58%	R.LALFPGVA R.DGEEAGAYDGPR.T	R.KTFSHELSDFGLESTAGEIPVVAIR.T	K.FVMQEEFSR.D
Protein disulfide-				LLLAAAR.L	K.GEKFVMQEEFSR.D	R.DGKALER.F
isomerase A3				R.LAPEYEAAATR.L	K.VVVAENFDEIVNNENK.D	R.FLQDYFDGNLK.R
				K.YGVSGYPTLK.I	K.DPNIVIAK.M	K.SEPIPESNDGPVK.V
				K.KQAGPASVPLR.T	K.MDATANDVPSPYEVR.G	R.VMMVAKK.F
				K.QAGPASVPLR.T	R.GFPTIYFSPANK.K	K.KFLDAGHK.L
				K.KFISDK.D	R.EATNPPVIQEEKPK.K	K.FLDAGHK.L
				K.DASIVGFFDDSFSEAHSEFLK.A	K.KAQEDL.-	K.LNFAVASR.K
				K.AASNLRDNYR.F	R.TEEEFK.K	K.KYEGGR.E
				K.DLLIAYYDVDYEK.N		R.ELSDFISYLQR.E
PDIA6_HUMAN	Q15084	48,490/4.95	22%	K.NRPEDYQGGR.T	K.FALLKGSFSEQGINEFLR.E	K.GESPVDYDGGR.T
Protein disulfide-				R.TGEAIVDAALSALR.Q	K.GSFSEQGINEFLR.E	K.MKFALLK.G
isomerase A6				K.LAAVDATVNQVLASR.Y	R.GSTAPVGGGAFPTIVER.E	R.ELSFGR.G
				R.EPWDGR.D		
PHB_HUMAN	P35232	29,843/5.57	40%	-.MAAKVFESIGK.F	K.AAIISAEGDSK.A	R.FDAGELITQR.E
Prohibitin				K.VFE R.AVIFDR.F	R.KLEAAEDIAYQLSR.S	K.EFTEAVEAK.Q
				SIGK.F	K.DLQNVNITLR.I	K.QVAQQEAER.A
				R.IFTSIGEDYDER.V	R.ILFRPVASQLPR.I	
TAGL2_HUMAN	P37802	22,548/8.41	44%	K.QM EQISQFLQAAER.Y	R.DDGLFSGDPNWFPK.K	K.NVIGLQMGTNR.G
Transgelin-2				R.YGINTTDIFQTVDLWEGK.N	R.NFSDNQLQEGK.N	
				R.TLMNLGGLAVAR.D	R.GPAYGLSR.E	
TBA1B_HUMAN	P68363	50,804/4.94	39%	R.AVFVDLEPTVIDEVR.T	R.IHFPLATYAPVISAEK.A	K.EIIDLVLDR.I
Tubulin α-1B chain				K.EDAANNYARGHYTIGK.E	R.HGKYMACCLLYR.G	R.LSVDYGK.K
				R.NLDIERPTYTNLNR.L	K.YMACCLLYR.G	K.CDPRHGK.Y
				R.FDGALNVDLTEFQTNLVPYPR.I	K.DVNAAIATIKTK.R	R.LISQIVSSITASLR.F
				R.AFVHWYVGEGMEEGEFSEAR.E	R.AVCMLSNTTAIAEAWAR.L	K.EDAANNYAR.G

Table S1. *Cont.*

Name	Accession Number	M_W (Da)/pI	Sequence cov. (%)	Peptides		
TBB5_HUMAN Tubulin β chain	P07437	50,095/4.78	52%	R.EIVHIQAGQCGNQIGAK.F	R.LHFFMPGFAPLTSR.G	R.GSQQYR.A
				R.ISVYYNEATGGK.Y	R.ALTVPELTQQVFDAK.N	K.NMMAACDPR.H
				R.AILVDLEPGTMDSVR.S	K.NSSYFVEWIPNNVK.T	R.YLTVAAVFR.G
				R.SGPFGQIFRPDNFVFGQSGAGNNWAG	K.TAVCDIPPR.G	R.GRMSMK.E
				K.GHYTEGAELVDSVLDVVR.K	.ISEQFTAMFR.R	R.FPGQLNADLR.K
				K.IREEYPDR.I	R.KLAVNMVPFPR.L	R.FPGQLNADLRK.L
				K.LTTPTYGDLNHLVSATMSGVTTCLR.F	K.LAVNMVPFPR.L	R.EEYPDR.I
TSG6_HUMAN Tumor necrosis factor-inducible gene 6 protein	P98066	31,204/6.3	10%	K.IGFHVCAAGWMAK.G		
				K.YVAMDPVSKSSQGK.N		
TPM4_HUMAN Tropomyosin α-4	P67936	28,619/4.67	46%	M.AGLNSLEAVKR.K	R.AEVSELK.C	K.YSEKEDK.Y
				R.ELDGER.E	K.NVTNNLK.S	K.LKEAETR.A
				R.RIQLVEEELDR.A	K.SLEAASEK.YT	R.AEFAER.
				R.KLVILEGELER.A	K.YEEVAR.K	R.LATALQK.L
						K.AADESER.G
TXD12_HUMAN Thioredoxin domain-containing protein 12	O95881	19,365/5.24	21%	K.VHPEIINENGNPSYKYFYVSAEQVVQGMK.E	K.KHLEDEL.-	
				R.LTGDAFR.K	R.TLEDGKK.E	
